# Automatic Field Detection of Western Corn Rootworm (*Diabrotica virgifera virgifera*; Coleoptera: Chrysomelidae) with a New Probe

**DOI:** 10.3390/insects11080486

**Published:** 2020-08-01

**Authors:** Zsolt Tóth, Miklós Tóth, Júlia Katalin Jósvai, Franciska Tóth, Norbert Flórián, Veronika Gergócs, Miklós Dombos

**Affiliations:** 1 Institute for Soil Sciences and Agricultural Chemistry, Centre for Agricultural Research, Herman Ottó út 15, H-1022 Budapest, Hungary; franciska.toth19@gmail.com (F.T.); florian.norbert@agrar.mta.hu (N.F.); gergocs.veronika@agrar.mta.hu (V.G.); dombos.miklos@agrar.mta.hu (M.D.); 2 Plant Protection Institute, Centre for Agricultural Research, Herman Ottó út 15, H-1022 Budapest, Hungary; toth.miklos@agrar.mta.hu (M.T.); josvai.julia@agrar.mta.hu (J.K.J.)

**Keywords:** agricultural monitoring, infrared sensor, invasive species, maize, pest management

## Abstract

The Western corn rootworm (WCR), *Diabrotica virgifera virgifera* LeConte (Coleoptera: Chrysomelidae), is a significant invasive pest of maize plantations in Europe. Integrated pest management demands an adequate monitoring system which detects the activity of insects with high accuracy in real-time. In this study, we show and test a new electronic device (ZooLog KLP), which was developed to detect WCR in the field. The ZooLog KLP consists of a trapping element that attracts insects with its color and species-specific sex pheromone. The other part is an opto-electronic sensor-ring which detects the specimens when they fall into the trap. At detection, the time of catch is recorded and sent to a web interface. In this study, we followed WCR flight patterns for six weeks in two locations, using ZooLog KLP probes. We investigated sensor precision by comparing the number of catches to the number of detections. The tool reached high accuracy (95.84%) in recording WCR. We found a peak in flight activity in August and a bimodal daily pattern. This method may be beneficial in detecting the WCR during their activity, and this new device may serve as a prototype for real-time monitoring systems and improve the management of this pest.

## 1. Introduction

Management of arthropod pests has long been a major challenge in food production for farmers throughout the world. Integrated pest management (IPM) programs [1] can achieve long term pest control with effective actions, such as monitoring the population size, estimating economic threshold, and integrating currently known chemical, biological, and physical control methods [2,3]. Therefore, the detection and identification of invertebrates are prerequisites necessity for IPM to optimize plant protection actions in agricultural fields.

Historically, different trap types have been applied for the detection and monitoring of pest species. However, most of them suffer from a number of shortcomings. For instance, during monitoring, traps should be checked at least weekly, with a visual count of the captures. Manual checking is a time-consuming and laborious task, especially in large agricultural fields. Moreover, data are delayed in time. Many initiatives approach the problem of manual checking by automatically enumerating the insects [4]. The accurate forecasting of potential pest outbreaks requires real-time detection of the insects in the field. Based on this information, pest control practices, such as selective spraying, can be arranged at the right time and location.

The Western corn rootworm (WCR), *Diabrotica virgifera virgifera* LeConte (Coleoptera: Chrysomelidae), ranks among the most critical invasive pests in Europe. It first appeared in Europe in the early 1990s [5], and currently infests most of the EU countries [6]. Larvae of WCR cause the primary damage to a maize plant by chewing and boring through the rootstock in the soil. However, beetles can also generate severe loss by feeding on the reproductive parts of the plant. The economic costs resulting from yield losses and pest management annually exceed 1 billion USD in the United States [7]. In the EU-27, potential damage to maize was estimated to be as high as 580 million USD per year, should all maize production areas be infested with WCR [8].

Protection against WCR mainly consists of cultural and chemical control. The cultural control methods, crop rotation, and delayed planting have been tested and modeled [9,10]. Annual rotation seems to be only partly effective, as the beetles have adapted to the rotation cycle (e.g., soybean *Gylcine max* L. in North America) [11,12]. As for the most common chemical control strategies, soil-applied insecticides and insecticide seed dressing are used against the larvae at planting [13]. Soil-applied insecticides showed a relatively higher efficacy to avoid root damage; however, insecticide application needs to be based on an effective monitoring program, as has been proposed for decades in many IPM programs (e.g., in the EU: Directive, 2009/128/EC). The abundance of adults on traps is used to identify population economic thresholds (e.g., 5–6 WCR adults/sticky trap/day in several growing areas).. The continuous and accurate counts of WCR over the season could be achieved more efficiently by automatic devices.

Foliage insecticide treatments are sometimes applied for WCR adults, to reduce population sizes and subsequent oviposition by females, or to avoid silk-clipping damage to maize silk. For these applications, it is necessary to know the current WCR population size; real-time, automatic measurement can also help. Annual flight patterns of WCR greatly vary according to locality, climate, and even years [14,15]. Adults generally occur from mid-July to mid-September, with a peak from the end of July to August [16,17]. The daily flight of WCR has a bimodal pattern, having two periods of maximal activity: some hours after sunrise and before sunset [18,19,20].

In Europe, two main types of traps are used to monitor WCR adults in the field, both containing female sex pheromone: yellow sticky traps and transparent sticky “cloak” traps called PAL (sticky sheet is shaped like a cloak, named after the Hungarian vocalist “Palást”) [21]. As beetles are attracted to yellow color, yellow sticky traps (e.g., Pherocon AM) are widely used, especially in North America. However, this trap type is more effective in areas where WCR populations are well established [22]. At low-population sites, PAL traps (CSALOMON^®^ PAL, Plant Protection Institute HAS, Budapest, Hungary) are more sensitive to detect the immigrant WCR population [23,24]. One issue with sticky traps is that non-target insects can quickly saturate them. To solve the problem of saturation, Tóth et al. [25] developed a non-sticky funnel-trap (so-called KLP “hat” trap) to catch WCR. This trap type has much higher catch capacity (5–6000 beetles compared to the 3–400 beetles in sticky traps), and is more user friendly than sticky traps.

For automatic measurement, sticky traps equipped with cameras recognize and also count dead or stuck insects in real-time, or with a relative time-lapse [26,27]. This type of machine vision technique yields an exact number of captured insects [28]; however, machine vision is still restricted only to sticky traps.

Although traps have been developed for the real-time, automatic detection of crawling or flying insects [see in 4], an automated detection technique for WCR has not yet been developed. In the case of the KLP trap, using photography is hardly possible; therefore, flying or falling insects have to be detected. For flying insects, opto-electronic devices such as those using laser beam [29] and infrared light [30,31] are common. In the case of these sensors, although good results have been achieved in distinguishing different species flying into the traps, for example, based on wingbeat frequency, e.g., [32], the problem is usually that most of the sensors are not species-specific.

Our team developed opto-electronic sensors, which automatically detect and count arthropods of different sizes [33]. These sensors effectively detect arthropods living in the soil or on the surface [34]. For agricultural pest forecasts, we equipped different pheromone traps with our sensors to achieve higher species specificity. The funnel traps (KLP trap) mentioned above are modified to automatically detect and count WCR and provide online access to those data. In this study, we provide a detailed technical description of our new probe. We present the precision and reliability of the new method by comparing the number of WCR individuals caught and detected by the ZooLog KLP probes under field conditions.

## 2. Materials and Methods

### 2.1. Description of the New ZooLog KLP Probe

The new probe was developed to catch and automatically count WCR specimens and forward those data to a central database. It comprises three main parts: a trap, a sensor, and a data communication system.

#### 2.1.1. Description of the Trapping Element

**Figure 1:**
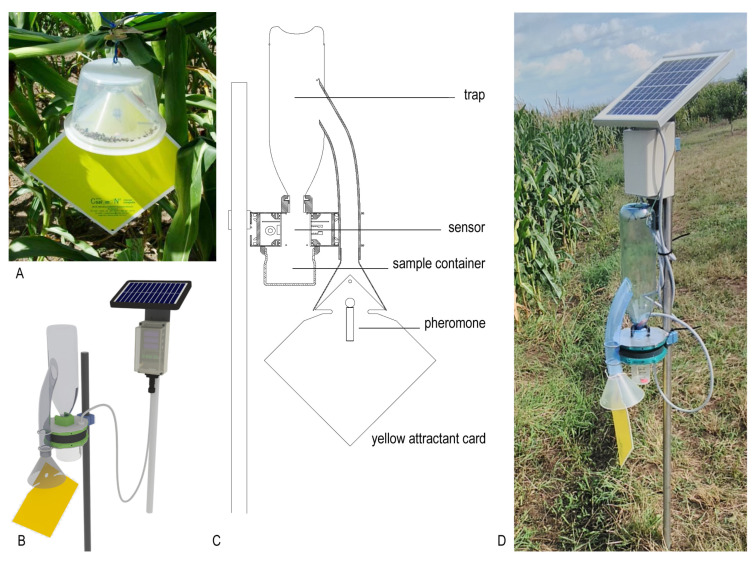
Construction of the new ZooLog KLP probe. (**A**): the original CSALOMON^®^ KLP “hat” trap. Western corn rootworm (WCR) individuals are attracted by pheromone towards an inverted funnel, from where they cannot escape. (**B**,**C**): the design and cross section of the new probe. The yellow attractant card and the inverted funnel are connected to a PET bottle, from where WCR individuals fall through the sensor-ring into the sampling container. Detection data are transferred to the central database via the internet. A solar panel provides power for the electronics, enabling online monitoring throughout the season. (**D**): The new probe deployed adjacent to the test cornfield.

The trapping element of our new probe is based on the CSALOMON^®^ KLP+ “hat” trap (Figure 1), produced by the Plant Protection Institute, Centre for Agricultural Research, Budapest, Hungary [25]. This trap type is typically used for quantitative sampling of WCR in Europe and can be baited with lures containing a species-specific sex pheromone or a floral-based attractant [35]. In our test, traps baited with the commercially available sex pheromone (CSALOMON^®^ group, Plant Protection Institute CAR, Budapest, Hungary) were used. The trap consists of a yellow, non-sticky plastic sheet panel, which attracts WCR with its color and an attached pheromone bait. The panel guides climbing WCR individuals through a funnel and a plastic tube towards a transparent plastic (PET) bottle (30 cm high with 8 cm diameter). The catch container should be transparent, because this species is attracted to light (positive phototaxis) [36]. The infrared (IR) sensor-ring (see next section) is mounted to the mouth of the PET bottle. The beetles spend approx. thirty minutes in the plastic bottle when first trapped, then eventually fall through the mouth of the bottle to the sensing unit. We used insecticidal strips (VaporTape, Hercon Environmental, Emigsville, PA, USA) to kill captured WCR adults, making manual counting of captured insects more convenient. The sensor-ring detects this event, and a sample container positioned in the lowermost part of the probe catches the specimens.

#### 2.1.2. IR Sensor-Ring

We used the uniform IR sensor-ring, which was developed by our team and has been optimized for the detection of insects with different body sizes [33]. The sensor part contains 16 pieces of IR sensors shifted in two rows for better coverage of the detection area and focused on the receiver. This opto-electronic sensor-ring records those events that interrupt the path between the receiver and the emitter. We constructed this sensor to detect arthropods falling or flying through the sensor field, and tested the precision and accuracy of its detection under laboratory conditions for several insect species. In the case of WCR, the detection accuracy was 100% with dead individuals (see Table 1 in [33]). According to this result, the sensor itself accurately detects WCR under stable conditions. However, the situation is entirely different under field conditions. In a noisy environment, raw detection data should be filtered to enhance precision and accuracy. For this procedure, we used artificial neural network (ANN) algorithms (see Section 2.3.1).

#### 2.1.3. Data Collection

**Figure 2:**
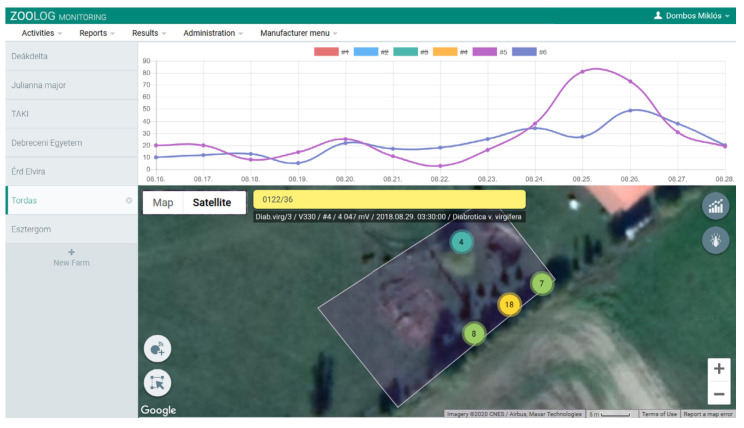
The ZooLog Online Web Interface: The monitoring system provides daily data via the internet. The probes and their status are visualized on a map. This interface is also used for setting the probes and data filtering. Results of the detection data can be downloaded and automatically visualized as graphs, typically the number of detected insects per day.

The ZooLog Monitoring system has been designed for online monitoring, including loggers, a central database, and a web interface. Data are transmitted to the central database each day (settings). Detection data can be downloaded from or directly managed on the ZooLog Online Web Interface (Figure 2). Obtained data can be reported according to the measurement series.

### 2.2. Field Observations

To estimate the precision and accuracy of the detection of the proposed sensor system, we conducted field surveys over six weeks, from the middle of August 2018 to the beginning of October 2018. A total of five probes were used; three probes were installed in cropland at Érd-Elvira Major Experiment Station of Research Institute for Fruit growing and Ornamentals, and two probes were placed in a privately owned cornfield near Tordas village in Hungary (Table 1). While the probes captured WCR, they continuously detected these individuals. We counted the individuals at least twice a week, found in the sample container of the probes. These datasets were the basis of data validation; we simply compared the number of detections to the number of catches obtained for a given time interval from the same probe.

### 2.3. Filtering Detection Data and Data Analysis

#### 2.3.1. Data Filtering

**Figure 3:**
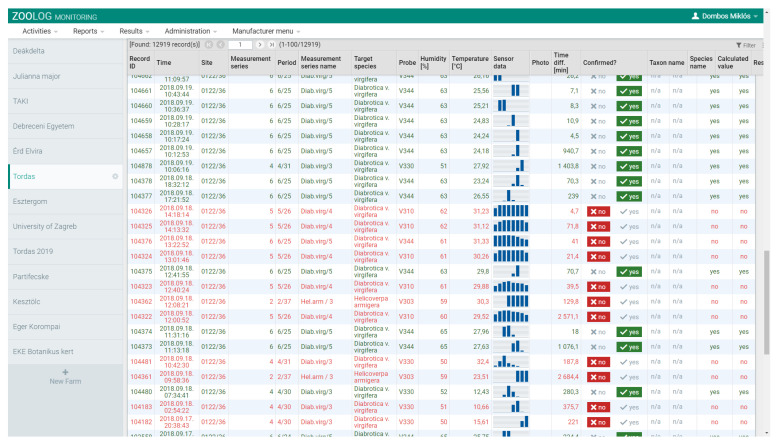
Filtering procedure for, and marking the records captured by, the ZooLog KLP probes. Each row corresponds to one detection. The sensor signal patterns are shown in the ‘Sensor data’ column. When only one or two signs were higher, we confirmed it manually in column ‘Confirmed?’ as “yes” (green colors) or “no” (red colors). We confirmed precisely the same number of detections that were captured in a given period. The patterns of positive detections differed significantly from the negative ones caused by other non-target insects. This procedure provided the learning dataset for deep-learning analysis used in filtering false detection data.

The IR sensor-ring provides a dataset at each detection, see [33]. Each detection consists of eight values generated by the sixteen IR sensors in the sensor-ring. The patterns of these raw output values are characteristic of WCR. WCR individuals usually triggered only one or two signals with higher figures due to their intermediate body size; therefore, we used these patterns to identify positive signals for WCR and filtered out noise data, e.g., signals generated by other non-target insects than WCR. For this procedure, as the main objective of field testing, we built up an interface in our database. In this operation table (Figure 3), one row corresponds to one detection, and the signal patterns appear in the column called ‘Sensor data’. During field tests, we checked and noted catches of each probe, at first hourly, and later daily. For the same periods, we listed the detection table on the interface. We tried to find the positive detections (caused by WCR) by eye, based on the characteristic signal patterns, and based on the numbers we caught in a given period. For example, if we find ten individuals in a period, we cannot be entirely sure exactly which ten signals belong to these individuals. We selected ten similar signals according to the characteristic signal patterns. This procedure formed the basis of the learning dataset, which was used in the machine learning process to allow automatic identification of positive signals. Deep-learning data analysis was performed with a large-scale distributed machine learning platform named TensorFlow [37], on Github [38]. The binary classification was built into the deep learning procedure (positive class: WCR individual), and the eight sensor figures were used as features for modeling. Ten percent of the dataset was labeled as examples in the learning procedure.

#### 2.3.2. Statistical Analysis

For the analyses, automatically detected and manually captured data were summed for each day per probe at both investigation sites (altogether five measurement series). For detection data, machine learning filtered data were used. Since both the distributions of captured and detected individuals per probe were strongly right-skewed (range: 1–421, median: 14), both the daily summed number of captures and detected individuals were ln (x + 1) transformed prior to the analysis.

The relationship between the automatically detected (and filtered) and the manually captured number of individuals was analyzed with a linear model to evaluate the accuracy, reliability, and overestimation rates of the sensor system. Daily data of the five probes were analyzed together. The intercept and the slope of the model were tested against 0 and 1, respectively, using the ‘multcomp’ package [39].

Several metrics (accuracy, precision, recall, and F1 score) were calculated to estimate the performance of the filtering procedure. In binary classification, according to the contingency matrix built up from the dataset, accuracy means true positives plus true negatives over the total number of examples. Precision is defined as the number of true positives over the number of true positives plus the number of false positives. Recall is defined as the number of true positives over the number of true positives plus the number of false negatives. These quantities are also related to the F1 score, which is defined as the harmonic mean of precision and recall. High precision is linked with a low false-positive rate, and high recall relates to a low false-negative rate.

Based on data derived from the automatic detection, some examples were presented to estimate and visualize the local daily and temporal activity of the western corn rootworm. Local polynomial regression fitting (locally estimated scatterplot smoothing, LOESS) was applied for smoothing time-series data.

All statistical analyses were carried out with R 3.6.2 software [40].

## 3. Results

### 3.1. Accuracy and Performance of the Sensor System

To achieve higher detection accuracy, we filtered out false detections. The filtering-out results of automatic detections gained by deep-learning analysis are summarized in Table 2. For the five probes together, there were 3266 true positives, 3611 true negatives, 184 false positives, and 313 false negatives out of 7374 cases. The machine learning reached a very high precision (0.95), due to relatively few false positives and negatives. The recall and F1 score for the adults of *D. v. virgifera* reached 0.91 and 0.93, respectively. The accuracy reached 0.93 for the whole data clearing procedure.

**Figure 4:**
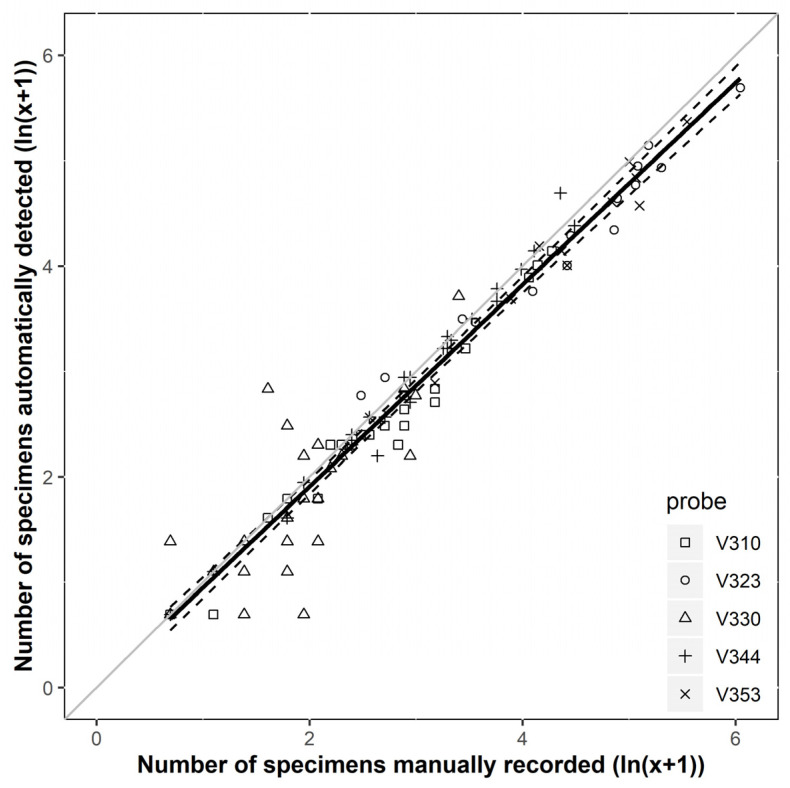
The relationship between the number of detections and captures of *D. v. virgifera* at Érd Elvira and Tordas sites using ZooLog KLP probes. One point represents the number of catches and the corresponding number of detections in a given period for a given probe. The solid line shows the predicted values from the linear model, and the broken lines show the 95% prediction interval. The solid grey line indicates the equality between the two variables.

Then, we compared the filtered detection data to the number of manually caught WCR specimens. Linear regression analysis showed a high correlation between automatic and manual counting after ln (x + 1) transformation (Figure 4). The average accuracy was 95.84%, with very high reliability (R^2^ = 0.948). The slope of the regression line was significantly lower than 1 (y = 0.96 ± 0.02, *p* < 0.001). However, there was no tendency for under- or overestimation of the number of individuals (intercept = −0.01 ± 0.07, *p* = 0.967).

### 3.2. Automatic Detection of WCR Over Time

**Figure 5:**
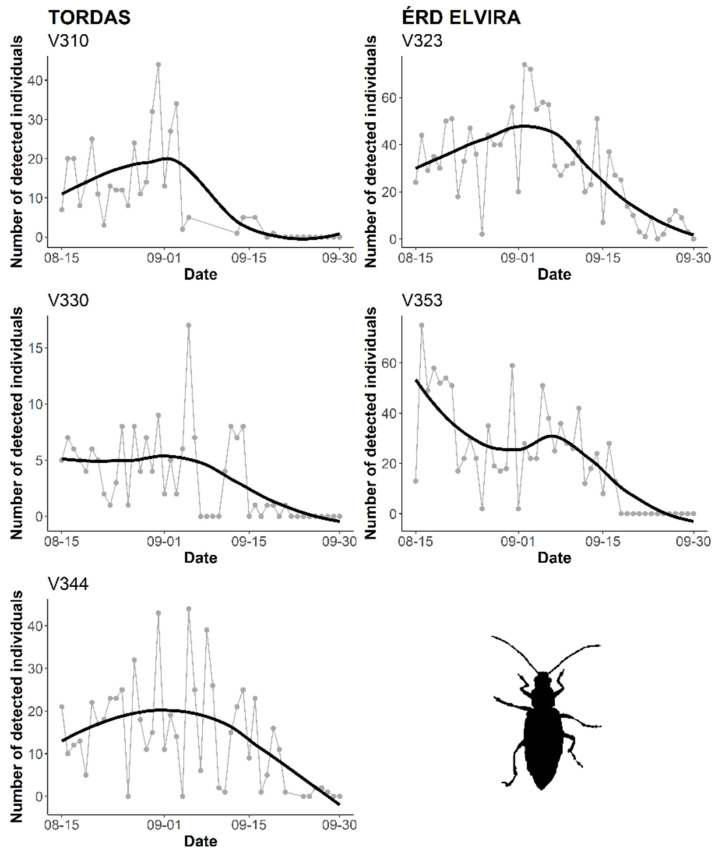
Continuous, automatic monitoring of *D. v. virgifera* by ZooLog KLP probes at Érd Elvira and Tordas research sites. For smooth curves, local polynomial regression fitting (locally estimated scatterplot smoothing, LOESS) was applied.

The inclusion of the sensor system in the trap allows us to follow the temporal activity of the target pest. Since our field tests were launched in August, the first half of the WCR flight activity was not captured. Preliminary results are presented to provide examples from monitoring WCR during the study period. Figure 5 illustrates the number of detected individuals obtained from probes placed at Érd Elvira (2) and Tordas (3) research sites. The temporal dynamics of the two data sets followed similar general trends, even if the curves of measurement series (probes) slightly varied. The automatic counting caught a small peak at the end of August/beginning of September (Figure 5).

**Figure 6:**
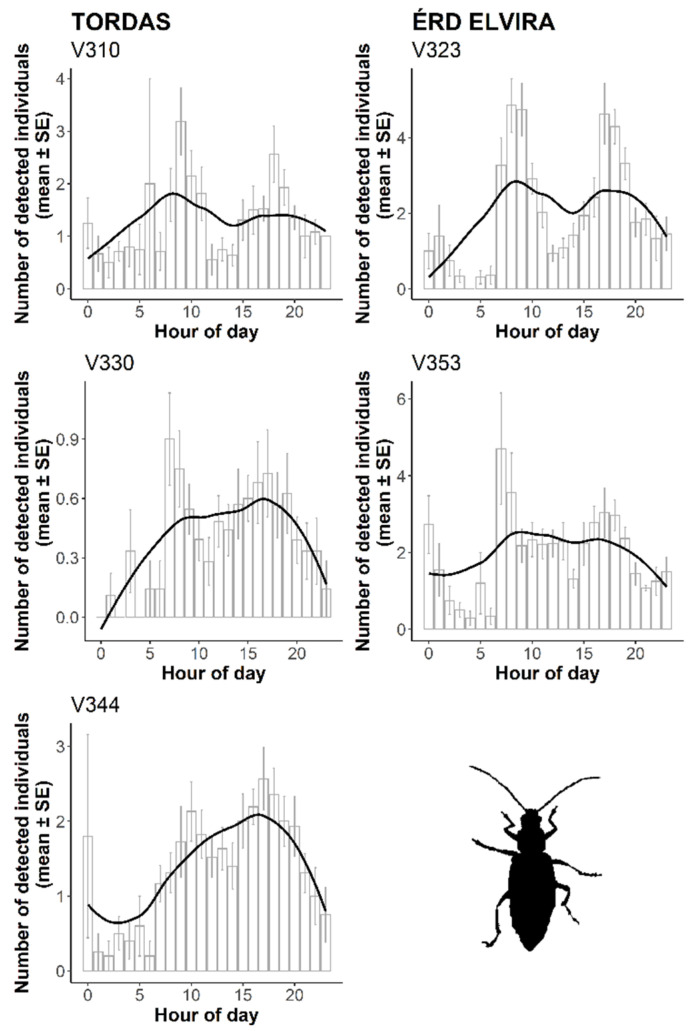
The activity circadian rhythms of *D. v. virgifera* based on probe recordings during six weeks from 15 August to 1 October 2018. For smooth curves, local polynomial regression fitting (locally estimated scatterplot smoothing, LOESS) was applied.

Employing the proposed sensor system, we estimated the local daily activity (circadian rhythm) of the Western corn rootworm. Combining all recorded events from the two research sites, WCR had periods of high activity twice a day. The daily peak periods were during the morning (between 8:00–9:00) and in the late afternoon (between 16:00–17:00) (Figure 6). The temporal pattern of data sets was similar in both sites, but the above-mentioned diurnal dynamic was more profound at the Érd Elvira site.

## 4. Discussion

The results of the present study clearly show that our new opto-electronic device (ZooLog KLP) can record WCR with high accuracy. In our previous laboratory tests with low environmental noise, the same sensor accurately detected (with 100% precision) several Coleoptera species larger than 1.40 mm, such as *Meligethes aeneus*, *Diabrotica virgifera*, *Agriotes ustulatus*, *Epicometis hirta*, *Cetonia aurata* [33]. Under semi-natural conditions, the sensor also detects small microarthopods (0.47–2.47 mm) with 88–100% accuracy, depending on species [41]. Compared to that, we reached 95.84% accuracy on average for WCR under field conditions.

For agricultural pest forecasting systems, species specificity of traps is essential. Several species-specific methods are used in the field to detect and recognize insect species automatically. Camera traps with machine vision [42,43,44,45] or using color sensors [46] have high species-specificity. However, these are useful only for sticky traps in which individuals caught can be easily photographed; photographing insects flying into the trap remains a technical challenge. Other devices recognize species according to their sound or wingbeat frequency using laser light [32] or stereo-recording [47]. Opto-electronic sensors are not able to reach species specificity; however, by using our infrared sensor-ring, we could select the positive signals of the target species (WCR) according to the body size. We were able to filter out the noise detections, which occurred in the same amount as the true-positive signals. False identifications, i.e., where the system identified WCR when the detection was not caused by WCR or vice versa, were rare; therefore, both the precision, accuracy and recall were very high, each of them was higher than 0.93. Sex pheromones are highly species-specific, and they are widely used in pest monitoring [48]. We equipped different types of the CSALOMON trap family with our sensors to detect flying insects (mostly moth species), click beetles [49], and also WCR. The ZooLog KLP trap combines the advantages of the attractiveness of the sex pheromone and the vertical climbing behavior of WCR [25], thus excluding most non-target species.

Traps baited with sex pheromone lures have the inherent disadvantage that they attract and catch only one sex (in the case of WCR, the males). Possible bias due to this in the interpretation of captures is widely discussed [48]. In the case of WCR, it is also possible to apply a synthetic floral lure [50], which acts as a feeding attractant and will attract both females and males [25]. By using the floral lure, the sex bias could be overcome. Our new trap device could be used with this lure; however, since a floral lure would attract other species more intensively than sex pheromones, the validation of the machine learning algorithm would likely need to be updated.

The advantage of our probe compared to sticky traps equipped with a camera is that it cannot easily be saturated. Since the system has low energy consumption that can be supplied by a small solar panel, our device can operate in remote sites for a long period without human intervention. Finally, cellular data transmission allows users to see online what is happening in the field.

To calculate action thresholds for *D. v. virgifera*, various characteristics have been used, such as the average and cumulative numbers of adults per plants [51]. Beyond qualitative monitoring and detection, pheromone traps can be best exploited in IPM when quantitative aspects of populations can be correlated to trap capture data. One approach is the establishment of threshold catch values connected with population densities or damage levels. Such a correlation study is very time consuming and should involve parallel experiments in several fields throughout the years. We believe that our new probe, due to its more efficient data acquisition and evaluation, will assist plant protection experts in performing such studies in the future. At present, to the best of our knowledge, no thresholds have been reliably established for any trap designs baited with the pheromone of WCR. Action thresholds calculated from different monitoring methods are not the same (e.g., for yellow sticky traps, they are lower than for pheromone traps) [24]. Moreover, in connection with WCR, even crop type affects the action threshold (e.g., the silage action threshold is lower than that for seed production or sweet corn) [13]. Future studies are needed, for example, to compare the performance of commercially used WCR traps with ZooLog KLP, to estimate the action threshold for this new device.

ZooLog KLP provides a new way of studying WCR population dynamics in the field. Flight of WCR starts in July, and adults are usually present until early October [15,16,17,52]. Since it gives the precise moment of each capture, creating a fine-scale data set compared to manual counting, circadian rhythms of WCR can be precisely investigated. All the studies investigating the daily flight activity of WCR, whether in the laboratory [19,53] or the field [18,20,54], and found that WCR has two flight peaks a day: in the morning and the evening. Similarly, in our study, captures also had maximal numbers between 8:00–9:00 and between 16:00–17:00. These monthly and daily patterns are usually correlated with climatic properties of the area [14,54]. Therefore, the detailed and accurate data gained by the ZooLog KLP may help to reveal the coherence between WCR flight timing and other variables. Unfortunately, our test was conducted only from the second half of the WCR flight period; therefore, we were not able to trace the whole seasonal pattern of WCR. More experimentation is needed on the entire beetle activity period to be able to evaluate trap efficacy and seasonal dynamics. However, ZooLog KLP detected the data in real-time. Most of the studies using trapping methods obtain data weekly, providing less sensitive analyses compared to the real-time approach. This detailed information may be beneficial when climatic and other variables are compared with activity data of pest insects.

The cost of the ZooLog KLP probe can only be roughly estimated as it is still in the prototype stage of development. To build the prototype, we used electronic and mechanical parts, which cost 288 Euros. The electronic board containing the infrared sensor-ring was custom designed and manufactured that led to a higher price (159 EUR). The very cheap looking infrared technology might provide an opportunity for a favorable cost-benefit ratio since, in serial production, the cost of such a board should not be higher than 25 EUR. The housing was made by 3D printing, had a fee of 65 Euros per probe. The output sensor datasets to be transferred via the internet are very small; therefore, considering the expense of online monitoring for one probe, the smallest prepaid data SIM card was enough for the whole year. Financial benefits for the growers could be the decrease in the number of on-site inspections of WCR. Since we can expect that real-time pest monitoring could contribute to reducing environmental impact, in the long run, the use of a more environmental-friendly technology could also lead to a business advantage.

## 5. Conclusions

Accurate and automatic monitoring methods are needed to achieve precise and effective pest control. This study is the first documented report of the ZooLog KLP as a continuous, automatic, real-time detection device to monitor WCR flight in the field. The ability to continuously and automatically measure WCR flight with high precision allows for more precise and, therefore, effective pest management of this species.

## Figures and Tables

**Table 1:** The geographical location of the test sites and ZooLog KLP probes.

Location	Description	Probe	Latitude	Longitude
Tordas	cornfield in a privately owned farm	V310	47°20′54.15″ N	18°43′58.65″ E
		V330	47°20′54.43″ N	18°43′59.22″ E
		V344	47°20′54.31″ N	18°43′58.95″ E
Érd Elvira	cornfield adjacent to an orchard	V323	47°20′23.89″ N	18°51′53.44″ E
		V353	47°20′24.91″ N	18°51′52.71″ E

**Table 2:** Contingency table showing the performance summary of the sensor system tested at 5 field sites in Tordas and Érd in 2018. ANN = artificial neural network, TP = true positive, TN = true negative, FP = false positive, FN = false negative.

		Automatically Detected (ANN Predicted)		
		1 (Positive)	0 (Negative)	Sum
**Manually Counted**	1	3266 (TP)	313 (FN)	3579
	0	184 (FP)	3611 (TN)	3795
	Sum	3450	3924	7374
